# Cost Implications of Improving Malaria Diagnosis: Findings from North-Eastern Tanzania

**DOI:** 10.1371/journal.pone.0008707

**Published:** 2010-01-14

**Authors:** Jacklin F. Mosha, Lesong Conteh, Fabrizio Tediosi, Samwel Gesase, Jane Bruce, Daniel Chandramohan, Roly Gosling

**Affiliations:** 1 National Institute for Medical Research, Mwanza, Tanzania; 2 London School of Hygiene and Tropical Medicine, London, United Kingdom; 3 Swiss Tropical Institute, Basel, Switzerland; 4 Università Bocconi, Milan, Italy; 5 National Institute for Medical Research, Tanga, Tanzania; New York University School of Medicine, United States of America

## Abstract

**Background:**

Over diagnosis of malaria contributes to improper treatment, wastage of drugs and resistance to the few available drugs. This paper attempts to estimate the rates of over diagnosis of malaria among children attending dispensaries in rural Tanzania and examines the potential cost implications of improving the quality of diagnosis.

**Methodology/Principal Findings:**

The magnitude of over diagnosis of malaria was estimated by comparing the proportion of outpatient attendees of all ages clinically diagnosed as malaria to the proportion of attendees having a positive malaria rapid diagnostic test over a two month period. Pattern of causes of illness observed in a <2 year old cohort of children over one year was compared to the pattern of causes of illness in <5 year old children recorded in the routine health care system during the same period. Drug and diagnostic costs were modelled using local and international prices. Over diagnosis of malaria by the routine outpatient care system compared to RDT confirmed cases of malaria was highest among <5 year old children in the low transmission site (RR 17.9, 95% CI 5.8–55.3) followed by the ≥5 year age group in the lower transmission site (RR 14.0 95%CI 8.2–24.2). In the low transmission site the proportion of morbidity attributable to malaria was substantially lower in <2 year old cohort compared to children seen at routine care system. (0.08% vs 28.2%; p<0.001). A higher proportion of children were diagnosed with ARI in the <2 year old cohort compared to children seen at the routine care system ( 42% vs 26%; p<0.001). Using a RDT reduced overall drug and diagnostic costs by 10% in the high transmission site and by 15% in the low transmission site compared to total diagnostic and drug costs of treatment based on clinical judgment in routine health care system.

**Implications:**

The introduction of RDTs is likely to lead to financial savings. However, improving diagnosis to one disease may lead to over diagnosis of another illness. Quality improvement is complex but introducing RDTs for the diagnosis of malaria is a good start.

## Introduction

Over diagnosis of malaria has been reported widely in both outpatient [1] and inpatient [2] settings using syndromic diagnosis [3] and with laboratory support [1], [2]. This is one of the reasons for the non recognition of the decreasing malaria transmission observed in sub-Saharan Africa, particularly in East Africa [4], [5], by global estimates of disease burden [6], [7], national statistics [8]and some researchers [9]. Over diagnosis of malaria not only contributes to the “scandal of ignorance of disease burden” but also to improper treatment leading to drug wastage and resistance, inefficient use of scarce resources, and most importantly poor health outcomes.

In Tanzania artemisinin-based combination therapy (ACT) is the 1^st^ line treatment for malaria. Microscopy or rapid diagnostic tests (RDTs) are used to confirm cases in health facilities that have these resources [10]. In places where diagnostic equipment or expertise is not available clinical diagnosis is the norm; this is a common practice in many parts of Africa particularly in primary health care facilities [11], [12].

There is a growing debate about the cost effectiveness of introducing RDTs in view of the adoption of expensive ACTs [10], [13], [14], [15], [16], [17] To date, costing and cost effectiveness models have focused on the cost of malaria diagnosis and treatment [15], [17], [18]. The few models that have estimated the costs of treatment of non-malaria fever diagnoses have used the cost of an antibiotic to represent this cost [10], [16], [19]. This paper attempts to estimate the rates of over-diagnosis of malaria in eight government health facilities in North-Eastern Tanzania and examines the potential cost implications of improving the quality of diagnosis in the context of a randomised controlled trial of different antimalarials for the prevention of malaria in infants (IPTi) [20].

## Methods

### Study Sites

**Figure 1:**
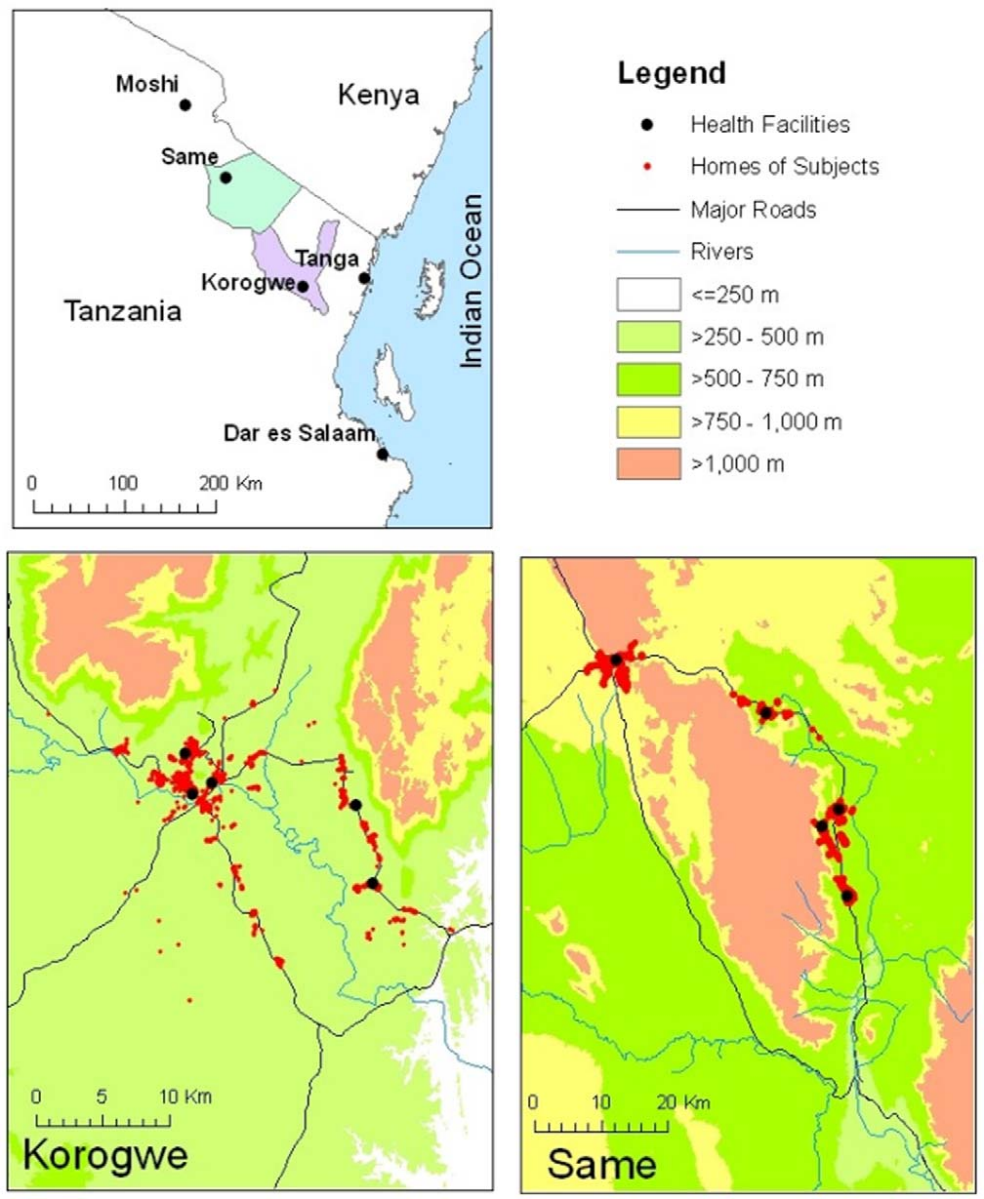
Study sites.

The study was conducted in a low and a moderate malaria transmission area in Tanzania. The low transmission site, Same, located in Kilimanjaro region (Figure 1, is semi-arid, with altitude ranging from 600m to 1000m in the plain and reaching up to 1900m in mountainous areas. Annual rainfall is low and seasonal, the main rains falling between March and June (mean annual rainfall 1996–2006 588ml range 265–1021ml). Incidence of malaria was recorded as 0.018 per child per year in children aged 2–24 months of age between 2005–2008 [20]. Health facilities included in the study were 1 district hospital in an urban setting (Same District Hospital), 1 health centre (Ndungu) and 2 dispensaries (Kiswani and Gonja Moare), situated in small rural townships.

The moderate transmission site, Korogwe District, Tanga region (Figure 1) is situated in the coastal plain with an altitude ranging from 200m to 600m. A small area of the district rises to above 1500m. This area was reported as holoendemic for malaria in 1991 [21] and has year round rainfall ranging from 800–1400ml. Incidence of malaria was recorded as 0.31 per child per year in children aged 2–24 months of age between 2005–2008 [20]. Health facilities included in the study in Korogwe were 1 peri-urban district hospital (Magnunga Hospital), 1 urban health centre (Majengo) and 2 rural dispensaries (Magazin and Mnyusi).

### Study Population for Estimating the Prevalence of Malaria

The magnitude of the over diagnosis of malaria was estimated by comparing data from 3 sources:

#### (i) Routine health information data

The causes of morbidity among the outpatient attendees (all age groups) from January to December 2007 were collected from the routine Health Management Information System (HMIS) registers kept at each health facility.

#### (ii) Health facility based cross sectional RDT survey

This component of the study was done in July to August 2007 in the 8 study health facilities. The study objectives were explained to all outpatient attendees (all age groups) requested to take part in the study. All patients attending for any illness and giving written consent were checked for malaria using an RDT (ParaHIT- *f*, Span Diagnostics, Surat, India,). The RDT results were reported to the health facility's clinical staff for making treatment decision.

#### (iii) Passive surveillance of causes of morbidity in a cohort of children

Data on causes of morbidity in 2 months to 2 year-old children who were enrolled in the IPTi study (reported elsewhere [20]) were collected through a passive surveillance system at the 8 study health facilities. For this analysis the causes of morbidity of children from the placebo groups from January to December 2007 was obtained from the IPTi study database. The IPTi study cohort was examined by study physicians on presentation to a health facility in the study area and all children with a history of fever were tested for malaria with an RDT and expert microscopy. A case of malaria was diagnosed only if the child had a history of fever in the previous 2 days or a recorded fever in the presence of parasitaemia as determined by 2 research microscopists. However, children who had a positive RDT were treated for malaria.

### Costing

The cost of treatment of morbidity pattern in <5 year old children based on the HMIS data of the year of 2007(data source (i) outlined above) was compared to that of the morbidity pattern observed in the IPTi study cohort (data source (iii) outlined above). The costing was undertaken from the perspective of the government health system and focused on the costs of diagnostic tools and drugs. Wider treatment costs to caretakers and providers associated with inpatient and outpatient care were not included in the analysis. Categories of cause of morbidity were based on those listed in the HMIS data and these were: malaria, acute respiratory infection, pneumonia, eye infection, ear infection, skin infections, urinary tract infection and ‘other’ (other is a mean cost of all other treatment groups). Treatment regimens and their associated costs were based on recommended first and second line treatment for a one year old infant weighing approximately 10kg, and the proportion of children likely to receive the various drugs was extrapolated from the prescribing practices of the study clinicians from the IPTi study cohort. For example, to determine the average unit cost of treating ARI, a weighted average was calculated to reflect the type and frequency of all the drugs given to all children identified and treated for ARI in the IPTi cohort. It was assumed that extrapolating the unit costs of treating illnesses based on the prescribing practices of the IPTi study clinicians would closely mirror the prescribing practice advocated by national treatment guidelines. Drug and diagnostic costs are modelled using local [22] and where these were not available, international, prices [23] and presented in 2007 US dollars.

The base case costing scenario assumed that in the IPTi study cohort all suspected cases of malaria received an RDT at a unit cost of US$0.75 [10] In the absence of data , it is assumed that 50% of all malaria diagnoses recorded in the HMIS data were identified using microscopy at a unit cost of USD0.27 [10] and the other 50% were treated presumptively. The cost of the ACT was US$0.95 [22] for both cohorts.

### Ethical Permission

Ethical permission was granted by the Ethical Review Boards of the National Institute of Medical Research of Tanzania and the London School of Hygiene and Tropical Medicine.

### Data Analysis

The percentage of outpatient attendees who were RDT positive and the percentage of attendees diagnosed as malaria in the HMIS data over the 2 month study period (July and August 2007) were compared in <5 and ≥5 year old age groups. The distribution of causes of morbidity in <5 year old children reported in the Routine Health care system data was compared to the distribution of causes of morbidity in the placebo group of the IPTi study cohort.

Potential cost savings by improving diagnosis were estimated by comparing the cost of treatment of causes of morbidity in <5 year old children reported by the Routine Health care system to the cost of treatment of morbidity observed in the placebo group of the IPTi study cohort. Results are presented as total cost to the public health care system (per 100 infants) and as ratio between IPTi cohort costs versus Routine health care system costs. Univariate sensitivity analysis was conducted on the main variables that were uncertain, prone to change over time and cost drivers. Specifically, the cost of the RDT was varied from US$1 to US$0.50, ACT costs from US$1 to US$0.50 and the rate of routine microscopy use from 100% to 0%.

## Results

### Over Diagnosis of Malaria

The comparison of the diagnosis of malaria confirmed by RDT and diagnosed clinically at the study health facilities are shown in Table 1. The magnitude of over diagnosis of malaria by the routine outpatient care system compared to RDT confirmed cases of malaria was highest among <5 year old children in the low transmission site (RR 17.9, 95% CI 5.8–55.3) followed by in the ≥5 year age group in the lower transmission site (RR 14.0 95%CI 8.2–24.2). The over diagnosis of malaria by the routine care system was high in the moderate transmission site as well though the magnitude was relatively low compared to the low transmission site (RR 2.3, 95%CI 1.8–3.0 in <5 year old children and RR 4.2, 95% CI 3.5–5.1 in ≥5 year age group).

**Table 1:** Comparison of RDT confirmed malaria vs. clinically diagnosed in routine health care system (July–August 2007).

HEALTH FACILITIES	UNDER 5 YEARS							OVER 5 YEARS						
	RDT SURVEY			Routine Health Care System			P- VALUE	RDT SURVEY			Routine Health Care System			P- VALUE
	N*	% positive for malaria	95% CI	N°	% diagnosed as malaria	95% CI		N*	% positive for malaria	95% CI	N°	% diagnosed as malaria	95% CI	
**KOROGWE**	***341***	**16.4**	***12.5–20.4***	***3122***	**38.1**	***36.4–39.8***	**P<0.001**	***804***	**11.7**	***9.5–13.9***	***4446***	**49.6**	***48.2–51.1***	**P<0.001**
**MAGUNGA**	*88*	19.3	*10.9–27.7*	*278*	51.4	*45.5–57.4*		*179*	10.6	*6.1–15.2*	*1594*	55.1	*52.6–57.5*	
**MAJENGO**	*91*	6.6	*1.4–11.8*	*1618*	37.2	*34.7–39.4*		*213*	2.8	*0.6–5.1*	*1424*	55.3	*52.7–57.9*	
**MAGAZINI**	*57*	29.8	*17.6–42.1*	*520*	34.8	*30.7–38.9*		*189*	18.5	*12.9–24.1*	*680*	37.9	*34.3–41.6*	
**MNYUZI**	*105*	15.2	*8.2–22.2*	*706*	37.8	*34.2–41.4*		*223*	15.2	*10.5–20.0*	*748*	38.0	*34.5–41.5*	
**SAME**	***300***	**1**	***0–2.1***	***2287***	**17.9**	***16.4–19.5***	**P<0.001**	***752***	**1.7**	***0.8–2.7***	***3154***	**24.3**	***23.0–25.5***	**P<0.001**
**SAME HOSP**	*51*	2.0	*0–5.9*	*1170*	21.4	*19.1–23.8*		*166*	1.8	*−2.4–3.8*	*2037*	15.9	*14.4–17.3*	
**KISIWANI**	*60*	1.7	*0–5.0*	*536*	0.0	*-*		*131*	2.3	*−0.3–4.9*	*536*	27.9	*24.8–31.1*	
**MAORE**	*90*	1.1	*1.1–3.3*	*182*	44.0	*36.7–51.2*		*216*	1.9	*0.04–3.7*	*182*	50.9	*46.7–55.1*	
**NDUNGU**	*99*	0	*-*	*399*	18.5	*14.7–22.4*		*239*	1.3	*−0.2–2.7*	*399*	26.5	*23.0–29.9*	

N* Number of patients tested with RDT.

**N**° Number of patients treated for malaria.

In the low transmission site, the variation in the proportion of patients having a RDT confirmed malaria did not vary substantially between the health facilities; it ranged from 0 to 2% in <5 year old children and 1–2% in ≥5 year old age group. However, the clinical diagnosis of malaria varied dramatically from 0–44% in <5 year old children and 16–51% in ≥5 year age group.

In the moderate transmission site, the variability in RDT confirmed and clinically diagnosed malaria between the health facilities was relatively low in both age groups except at Majengo dispensary which serves an urban population. At this urban health facility, the disparity between the RDT confirmed and clinically diagnoses malaria was high in <5 year old children (RR 5.6 95%CI 2.6–12.2) and in ≥5 years age group (RR 19.6 95%CI 8.9–43.2). The results from this urban dispensary are similar to those from the low transmission site.

A comparison of the distribution of causes of morbidity in the <2 year old children enrolled in the IPTi study cohort diagnosed by study clinicians [20]and in the <5 year old children in the same health facilities diagnosed by routine health care providers in the two sites in 2007 is shown in Table 2. In the low transmission site the proportion of morbidity attributable to malaria was substantially lower in the IPTi cohort compared to children seen at the routine care system. (0.08% vs 28.2%; p<0.001). A higher proportion of children were diagnosed with ARI in the IPTi cohort compared to the children seen at the routine care system ( 42% vs 26%; p<0.001). More children were diagnosed with urinary tract infection in the IPTi cohort than in the children seen at the routine care system (19% vs 3%; p<0.001).

**Table 2:** Comparison of causes of morbidity in <2 year old children in the IPTi study cohort and in <5 old children seen in the routine heath care system in 2007 in the study health facilities.

Diagnosis	KOROGWE (Moderate transmission site)				SAME (Low transmission site)			
	IPTi cohort		Routine health care system		IPTi cohort		Routine health care system	
	%	N	%	N	%	N	%	N
**MALARIA**	*9.8*	*147*	*44.3*	*6640*	*0.1*	*1*	*28.2*	*4679*
**ARI**	*39.4*	*592*	*18.4*	*2756*	*41.7*	*502*	*26.3*	*4261*
**PNEUMONIA**	*6.9*	*104*	*11.8*	*1768*	*8.7*	*105*	*14.4*	*2333*
**DIARRHOEA**	*9.2*	*138*	*6.7*	*1011*	*14.4*	*173*	*13.4*	*2163*
**EYE INFECTIONS**	*2.6*	*39*	*1.6*	*239*	*3.2*	*38*	*1.9*	*310*
**EAR INFECTIONS**	*1.2*	*18*	*1.4*	*215*	*1.5*	*18*	*0.9*	*143*
**SKIN INFECTIONS**	*9.2*	*138*	*3.1*	*461*	*7.7*	*93*	*1.6*	*315*
**UTI**	*14.9*	*224*	*8.3*	*1242*	*18.9*	*227*	*3.0*	*480*
**OTHER**	*7.0*	*105*	*4.4*	*656*	*3.9*	*47*	*0.7*	*118*
**TOTAL**	***100.0***	***1503***	***100.0***	***14988***	***100.0***	***1204***	***100.0***	***16184***

The same pattern was observed in the high transmission site. A higher proportion of children were diagnosed with malaria in the children seen in the routine care system compared to that in the IPTi cohort (44% vs 10%; p<0.001) and the proportion of children with a diagnosis of ARI was lower in the routine care compared to the IPTi cohort (18% vs 39%; p<0.001)

### Drug and Diagnostic Costs

The unit costs of treating each causes of morbidity are shown in Table 3. At US$1.92 and US$1.31 in the IPTi cohort and children receiving routine care respectively, the unit cost of treating a child with malaria was the most costly outpatient cause of morbidity to treat.

**Table 3:** Costing assumptions.

Categories of causes of morbidity and Drugs Prescribed	Estimated Proportion of children receiving this prescription	Cost of treatment per category of morbidity per child (US $ 2007)
**Malaria**		**1.92** a
		**1.31** b
(ALU) COARTEM	100%	
Paracetamol	100%	
RDT (IPTi group)	100%	
Microscopy (HMIS/routine group)	50%	
**ARI**		**0.59**
AMOXYCILINE SYRUP	76%	
Cotrimoxazole Syrup	11%	
Erythromycin Syrup	6%	
Chloromphenical	7%	
COUGH SYRUP	78%	
Paracetamol	44%	
**Pneumonia**		**0.90**
AMPICILLIN/GENTER	100%	
**Diarrhoea**		**1.02**
ORS	100%	
Ringer lactate intravenous fluid	100%	
Cotrimoxazole	48%	
Erythromycin	13%	
Amoxycillin	9%	
Ceftriaxone	6%	
Ampicillin and gentamycin injection	24%	
**Gastroenteritis**		**0.56**
ORS	100%	
Erythromycin Tabs	61%	
Ampicillin injection and gentamycin injection	8%	
Cotrimoxazole Syrup	22%	
Amoxycillin syrup	6%	
Metronidazole Syrup	3%	
**Eye Infection**		**0.72**
Tetracycline eye ointment	100%	
**Ear Infection**		**0.86**
Amoxycillin syrup	34%	
ampiclox syrup	74%	
Erythromycin Syrup	6%	
Boric acid/gentamycin ear drops	81%	
**Skin Infections**		**0.92**
G.V PAINT	54%	
Cloxacillin syrup	67%	
Ampiclox syrup	33%	
**Urinary Tract Infection**		**0.53**
Amoxycillin syrup	61%	
Chloromphenical	11%	
Ampicillin injection and gentamycin injection	18%	
Erythromycin	4%	
Cotrimoxazole	6%	
Paracetamol	100%	
**Other**		**0.89**
Cost per infant is an average of other intervention costs		

a Cost per infant – IPTi Cohort (all suspected cases of malaria received an RDT).

b Cost per infant routine health system (50% of all malaria diagnoses identified using a microscopy and the other 50% were treated presumptively).

The costs of treating each category of morbidity per 100 children based on the proportional morbidity in the IPTi cohort and children treated at the routine care system is presented in Table 4. In total costs, ARI was the most costly diagnostic category in both transmission settings in the IPTi cohort, whereas malaria was the most costly diagnostic category in both sites in the routine care system. Using a RDT (as was done 100% of the time in the IPTi study cohort) reduced overall drug and diagnostic costs by 10% in the high transmission site and by 15% in the low transmission site, when compared to total diagnostic and drug costs of treatment based on clinical judgment in the routine health care system. This was largely due to the reduction in malaria treatment costs by 66% and 99% in the moderate and low transmission sites respectively when an RDT was used to guide treatment compared to costs based on the clinical diagnosis of malaria. The total treatment costs for ARI, skin infections and UTI were notably higher in both transmission settings in the IPTi cohort compared to the children treated in the routine care group. The total costs of treating pneumonia were lower in the IPTi cohort compared to routine care group.

**Table 4:** Drug and diagnostic costs (per 100 infants treated).

Costs	KOROGWE		SAME		
	(Moderate transmission site)		(Low transmission site)		
	IPTi Cohort	Routine Health Care§	Cost Comparison*	IPTi Cohort	Routine Health Care	Cost Comparison
	Total Costs	Total Costs		Total Costs	Total Costs	
**MALARIA**	*18.85*	*57.95*	*33%*	*0.19*	*36.89*	*1%*
**ARI**	*23.38*	*10.92*	*214%*	*24.74*	*15.61*	*159%*
**PNEUMONIA**	*6.21*	*10.62*	*58%*	*7.83*	*12.96*	*60%*
**DIARRHOEA**	*9.39*	*6.84*	*137%*	*14.70*	*13.68*	*107%*
**EYE INFECTIONS**	*1.86*	*1.14*	*163%*	*2.29*	*1.36*	*168%*
**EAR INFECTIONS**	*1.03*	*1.20*	*86%*	*1.28*	*0.77*	*167%*
**SKIN INFECTIONS**	*8.49*	*2.86*	*297%*	*7.10*	*1.48*	*481%*
**UTI**	*14.90*	*4.38*	*340%*	*9.98*	*1.58*	*630%*
**OTHER**	*6.24*	*3.92*	*159%*	*3.90*	*0.62*	*625%*
**TOTAL**	***90.34***	***99.83***	***90%***	***72.03***	***84.95***	***85%***

§ Routine Health Care represents data from HMIS.

* IPTi cohort costs/Routine health care system costs.

**Figure 2:**
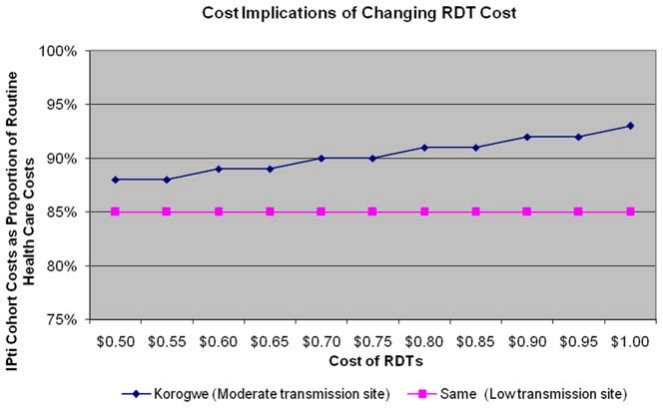
Sensitivity Analysis on cost implications. Figure 2a: Cost Implications on Changing the Cost of RDTs. Figure 2b: Cost Implications on Changing the Cost of ACT. Figure 2c: Cost Implications on Changing the Rates of Routine Microscopy.

Figure 2 shows the impact of changing the unit cost of a RDT and ACTs, and the rate of routine microscopy. At the low transmission site there was a 15% cost saving in the IPTi cohort irrespective of RDT costs varying from US$1 to US$0.50. This is to be expected as very few outpatient cases (0.19%) were RDT confirmed malaria. In the moderate transmission site, if RDTs were used, the subsequent correct diagnosis and treatment of children resulted in a 7 to 12% savings, when the cost of an RDT is varied from US$1 to US$ 0.50 respectively.

In the moderate transmission site, there were 7% savings to 15% additional costs if the cost of ACT varied from US$1 to US$ 0.50 per child treated respectively. The use of RDTs to improve the accuracy of malaria diagnosis was only cost saving if ACTs cost more than US$ 0.63. In the low transmission site, if the cost of ACT varied from US$1 to US$ 0.50 per child treated, there was a 17% savings to 7% additional costs respectively. RDTs were only cost saving if ACTs were more than US$0.50.

The frequency of use of microscopy in routine care system had a limited impact on cost savings. More importantly, however, even if rates of microscopy use varied from 100% to 0%, the use of RDTs in the IPTi cohort was always cost savings in both transmission settings when compared to the routine care system. In the moderate transmission setting, if microscopes were used 100% of the time to diagnose malaria in routine care then the RDT group showed 15% savings, if microscopes were never used, the IPTi cohort showed 4% cost savings compared to routine care system. In the low transmission settings the savings were between 19% (100% microscopy use in routine care system ) and 11% (no microscopy use).

## Discussion

Over diagnosis of malaria occurs at high rates in both low and moderate transmission settings. It is interesting to note that although malaria is less common in the low transmission setting and the risk ratio (RR) of over diagnosis is high, the actual numbers of those wrongly diagnosed as having malaria is greater in the moderate transmission setting as malaria is more frequently diagnosed. This over diagnosis of malaria occurs largely at the expense of under diagnosing ARI and pneumonia. This study shows that in both study districts malaria is significantly over-diagnosed at the primary health care level and that this over-diagnosis is massive in low transmission areas as well as in small urban settings (townships) such as district capitals in moderate transmission areas. The high burden of malaria was observed mostly in health facilities which were located in rural areas, and this important difference has not been detected by the routine care system. Rural communities bear the brunt of malaria burden. However, due to growing townships in rural areas this population at risk of malaria is decreasing. The population in small townships is now living with the trappings of modern urban life of improved housing, access to health care, access to education, higher population density and decreased malaria transmission [24].

Over diagnosis is costly in physical and financial terms. The physical cost is in people who are ill due to a cause other than malaria and not getting the appropriate treatment leading to severe disease and mortality [2]. Financially children that are misdiagnosed are likely to require subsequent consultations and receive additional treatment that may require more expensive drugs and inpatient care. This increases costs to both the households [25], [26]and health care providers [27]. We have shown that by improving the quality of diagnosis the burden of malaria is being reduced as non malaria illness are better diagnosed and treated, simply by improving accuracy, and an overall benefit on morbidity and mortality is possible. However there is a need to equip health facilities with important and simple diagnostic equipments in order to facilitate improvements in clinical care. In the IPTi study cohort 15%–20% of the children who attended a study health facility were diagnosed as having urinary tract infections. This is most probably a further over diagnosis of another cause of morbidity and relates to what clinicians do when they can no longer use malaria as a ‘catch all’ diagnosis. The next step in this case would be to introduce urine dipstick analysis. However, this would mean additional investment to the health infrastructure such as sterile pots to collect urine, a fridge to store the reagent strips, training in their use, etc., which would lead to additional costs.

Our cost analysis suggests that there is a thresholds where the price of an infant dose of ACT no longer makes RDT use cost saving. In the low transmission setting this is US$ 0.50 and in the moderate transmission setting this is US$ 0.63. If, however, the cost of ACT was to remain at US$0.95, even if the cost of the RDT were to increase to US$1 RDTs would still be cost saving. Regardless of the level of microscopy use in routine practice, RDT use would always be cost saving in both transmission settings. This is likely to be due to the low quality of routine microscopy [28] , the relatively easier interpretation of RDT results than reading a blood slide and less dependent on technical skills and resources. It is important to note, however, that we talk about ‘cost saving’ in terms of drugs and diagnostic costs only. If this cost analysis was broadened to include the cost implications of incorrect diagnoses incurred at both the household and health system level the likelihood is that RDTs would be cost-effective from the societal perspective even if the unit cost of an RDT was to increase beyond the thresholds identified here.

We have looked at the cost implications of using RDTs to better identify malaria episodes and in addition the costs associated with treating a range of non malaria illness episodes. The paper does not undertake a rigorous cost effectiveness analysis [10], [18] or cost benefit analysis [14] as seen elsewhere, therefore it would be inaccurate to compare the cost of this paper directly with analysis undertaken in other studies. However, certain findings in this study are supported by economic evaluations modelled elsewhere. Other models have demonstrated that locations where RDT use is preferable is dependent on factors such as malaria transmission intensity and the costs and accuracies of the RDTs under consideration [13].

In southern Mozambique the use of RDTs in all suspected cases of malaria was shown to be cost-saving when parasite prevalence among clinically diagnosed malaria cases was low to moderate. However, the study also suggested that targeting RDTs at the group older than 5 years and treating children less than 5 years on the basis of clinical diagnosis was even more cost-saving [17]. A model developed to illustrate the use of the two main classes of RDTs in the Ugandan context demonstrated that if only direct expenditures are considered, the pLDH test is the preferred option for adult patients except in high transmission settings, while young children are best treated presumptively in all settings. When health outcomes were considered, the HRP2 test gained an advantage in almost all settings and for all age groups [13]. A cost benefit study conducted in north-eastern Tanzania found that at high and low levels of malaria transmission, rapid diagnostic tests were more cost beneficial than microscopy, and both more so than presumptive treatment, but only where a clinicians prescribing response was consistent with test results [14]. The importance of health care provider behaviour in relation to test results was also found to greatly influence cost savings in Zambia [18] and Southern Mozambique. It is important to better understand the behaviour of health workers when using RDTs as they do not always comply with results, even if true negative [29] and this has cost implications [12].

### Limitations

The paper uses data from different sources and although we make direct comparisons, readers should consider the extrapolations that we have made. There are certain limitations when making comparisons between Routine Health care system and RDT survey data, as the Routine Health care system data contains the same people who are sampled in the RDT survey and the Routine Health care system data is known to be incomplete as not all attendees would have been registered. However, the Routine Health care system data is used for government statistics and its weaknesses have to be acknowledged and accepted [30]. When comparing the IPTi study cohort with the Routine Health care system, there is an age difference between comparison groups as the IPTi study cohort were under 2 years of age while the Routine Health care system included children <5 years. Children under 2 years are more likely to have more sickness than the under 5's and the distributions of illnesses may be different. There is also a possibility of having a trial effect in the IPTi study cohort. The IPTi study cohort (albeit placebo group) may have had less serious cases as they may have had different health seeking behaviour as they were encouraged to attend at the health facilities in the presence of any symptom. However, we have given an approximation of the likely proportions of disease burden that routine health care systems in rural districts in Tanzania faced in 2007. This will allow policy makers to readjust their assumptions on malaria burden and to understand that establishing disease burden is a moving target and requires more attention.

The costing part has its limitations. Most notably, we do not consider the effect of variability in the specificity and sensitivity of RDTs on the cost savings. The sensitivity and specificity of RDTs is reported to be 90% and 95% respectively compared with expert slide reading [1], [31], [32]. We assume that the sensitivity and specificity would be 100% for both RDTs and microscopy for the cost analysis. While we acknowledge the potential limited accuracy of some RDTs, particularly the low sensitivity of some RDTs, this is not incorporated into our analysis. It should be noted however, that given the low prevalence of malaria in both the study settings, the actual number of false negatives is likely to be extremely low, compared to the number of false positives in routine clinical diagnosis and the difference will continue to increase where malaria prevalence is falling. We also assume that the clinician prescribes on the basis of the test results. Previous research in Tanzania suggests this has not always been true in practice [29], [33]. Our costing perspective does not include the added saving from averting inpatient care, both from malaria and other causes of illness nor account for the added costs of false diagnosis. The costs are from the perspective of government funded health facilities. A previous study conducted in north-eastern Tanzania showed only 45% of under fives seeking care in these type of facilities (unpublished data). The cost savings from slowing drug resistance are outside the remit of the study [34]

In both the RDT survey and the IPTi study cohort [20], the prevalence of malaria was much higher in the two health facilities which were located in the rural areas compared to those located in small towns. In the HMIS report this was not observed and showed an equal distribution malaria cases in all the health facilities. Burden estimates tend to be based on extrapolation of prevalence of malaria observed in research sites to whole districts and where no information exists, from a nearby district [6]. This study highlights that even in areas of moderate transmission; estimates of malaria burden will be biased if it is assumed that the small townships have the same malaria risk as the rural populations.

There has been a decrease in malaria across the region which national statistics and clinical diagnoses have not reflected. In this paper we show that improving accuracy of diagnosis by using RDTs and with the benefit of continuous supervision by a research team, similar to continuous medical education (CME) widely adopted in developed countries, over diagnosis of malaria can be detected and appropriate treatments can be given to patients. RDT testing should be implemented nationally and a record of RDT positive diagnoses fed back to the National Malaria Control Program, where control activities could focus on the places where malaria is truly a problem. Health education about malaria and the alternative diagnoses needs to be directed at clinicians and in a more general way to the general population. The extent to which RDTs are cost saving will be associated with the frequency of their use. If the practice of over diagnosis of malaria is widespread, an initial over use of RDTs can be expected, which will carry additional costs. Overtime however, the frequency of true negative readings should go a long way to reassure health care providers and the general population that the prominence given to malaria is unfounded, and therefore RDT use in general should fall. Currently supervisory visits focus on preventive services especially EPI and more recently IMCI. We suggest that these visits should be broader and include supervision on curative services for all age groups and focus on improving diagnosis. This study sheds some light on what diagnoses health services should expect and suggests that respiratory tract disease and diarrhoea are still major causes of morbidity in Africa even in areas of high transmission of malaria.

### Policy Implications

Malaria transmission differs enormously throughout Tanzania [35] with the majority of the country being moderate and low transmission. Thus the introduction of RDTs is likely to lead to financial savings. However, improving diagnosis to one disease may lead to over diagnosis of another illness, in this case urinary tract infections; highlighting the need for ever more complex diagnostic and health services. Quality improvement is complex but introducing RDTs for the diagnosis of malaria is a good start.
