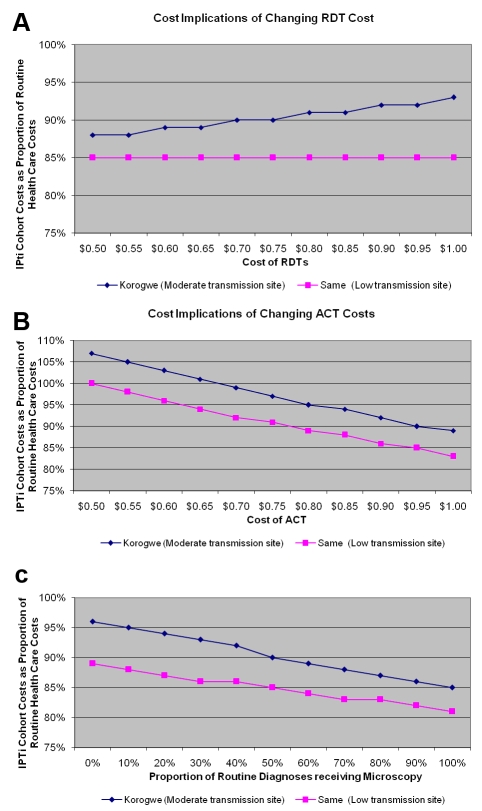# Correction: Cost Implications of Improving Malaria Diagnosis: Findings from North-Eastern Tanzania

**DOI:** 10.1371/annotation/84df39c4-a548-4214-af76-09068fdc2c4c

**Published:** 2010-01-25

**Authors:** Jacklin F. Mosha, Lesong Conteh, Fabrizio Tediosi, Samwel Gesase, Jane Bruce, Daniel Chandramohan, Roly Gosling

Figure 2 is missing a panel. Please view the correct version of Figure 2 here: 

**Figure:**